# Accelerating 3D Medical Image Segmentation by Adaptive Small-Scale Target Localization

**DOI:** 10.3390/jimaging7020035

**Published:** 2021-02-13

**Authors:** Boris Shirokikh, Alexey Shevtsov, Alexandra Dalechina, Egor Krivov, Valery Kostjuchenko, Andrey Golanov, Victor Gombolevskiy, Sergey Morozov, Mikhail Belyaev

**Affiliations:** 1 Center for Neurobiology and Brain Restoration, Skolkovo Institute of Science and Technology, 121205 Moscow, Russia; Alexey.Shevtsov@skoltech.ru (A.S.); m.belyaev@skoltech.ru (M.B.); 2 Sector of Data Analysis for Neuroscience, Kharkevich Institute for Information Transmission Problems, 127051 Moscow, Russia; Egor.Krivov@frtk.ru; 3 Department of Radio Engineering and Cybernetics, Moscow Institute of Physics and Technology, 141701 Moscow, Russia; 4 Moscow Gamma-Knife Center, 125047 Moscow, Russia; adalechina@nsi.ru (A.D.); VKostjuchenko@nsi.ru (V.K.); 5 Department of Radiosurgery and Radiation, Burdenko Neurosurgery Institute, 125047 Moscow, Russia; Golanov@nsi.ru; 6 Medical Research Department, Research and Practical Clinical Center of Diagnostics and Telemedicine Technologies of the Department of Health Care of Moscow, 127051 Moscow, Russia; gombolevskiy@npcmr.ru (V.G.); morozov@npcmr.ru (S.M.)

**Keywords:** deep learning, medical image segmentation, computed tomography (CT), magnetic resonance imaging (MRI)

## Abstract

The prevailing approach for three-dimensional (3D) medical image segmentation is to use convolutional networks. Recently, deep learning methods have achieved human-level performance in several important applied problems, such as volumetry for lung-cancer diagnosis or delineation for radiation therapy planning. However, state-of-the-art architectures, such as U-Net and DeepMedic, are computationally heavy and require workstations accelerated with graphics processing units for fast inference. However, scarce research has been conducted concerning enabling fast central processing unit computations for such networks. Our paper fills this gap. We propose a new segmentation method with a human-like technique to segment a 3D study. First, we analyze the image at a small scale to identify areas of interest and then process only relevant feature-map patches. Our method not only reduces the inference time from 10 min to 15 s but also preserves state-of-the-art segmentation quality, as we illustrate in the set of experiments with two large datasets.

## 1. Introduction

Segmentation plays a vital role in many medical image analysis applications [1]. For example, the volume of lung nodules must be measured to diagnose lung cancer [2], or brain lesions must be accurately delineated before stereotactic radiosurgery [3] on Magnetic Resonance Imaging (MRI) or Positron Emission Tomography [4]. The academic community has extensively explored automatic segmentation methods and has achieved massive progress in algorithmic development [5]. The widely accepted current state-of-the-art methods are based on convolutional neural networks (CNNs), as shown by the results of major competitions, such as Lung Nodule Analysis 2016 (LUNA16) [6] and Multimodal Brain Tumor Segmentation (BraTS) [7]. Although a gap exists between computer science research and medical requirements [8], several promising clinical-ready results have been achieved (e.g., [9]). We assume that the number of validated applications and subsequent clinical installations will soon grow exponentially.

The majority of the current segmentation methods, such as DeepMedic [10] and 3D U-Net [11], rely on heavy 3D convolutional networks and require substantial computational resources. Currently, radiological departments are not typically equipped with graphics processing units (GPUs) [12]. Even when deep-learning-based tools are eligible for clinical installation and are highly demanded by radiologists, the typically slow hardware renewal cycle [13] is likely to limit the adoption of these new technologies. The critical issue is low processing time on the central processing unit (CPU) because modern networks require more than 10 min to segment large 3D images, such as a Computed Tomography (CT) chest scan. Cloud services may potentially resolve the problem, but privacy-related concerns hinder this solution in many countries. Moreover, the current workload of radiologists is more than 16.1 images per minute and continues to increase [14]. Though slow background processing is an acceptable solution for some situations, in many cases, nearly real-time performance is crucial even for diagnostics [15].

Scarce research has been conducted on evaluating the current limitations and accelerating 3D convolutional networks on a CPU (see the existing examples in Section 2). The nonmedical computer vision community actively explores different methods to increase the inference speed of the deep learning methods (e.g., mobile networks [16] that provide substantial acceleration at the cost of a moderate decrease in quality). This paper fills this gap and investigates the computational limitations of popular 3D convolutional networks in medical image segmentation using two large MRI and CT databases.

**Figure 1:**
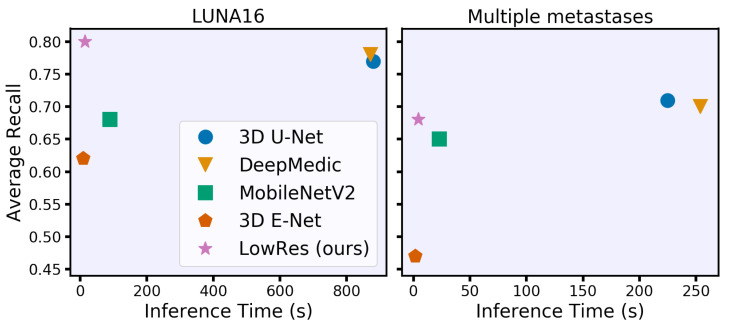
Time-performance trade-off for different convolutional neural network models under 8 GB of RAM and eight central processing unit thread restrictions. We evaluate models on two clinically relevant datasets with lung nodules (LUNA16) and brain metastases in terms of the average object-wise recall (LUNA16 competition metric [17]). Our model spends less than 15 s per study on processing time while preserving or even surpassing the performance of the state-of-the-art models.

Our main contribution is a new acceleration method that adaptively processes regions of the input image. The concept is intuitive and similar to the way humans analyze 3D studies. First, we roughly process the whole image to identify areas of interest, such as lung nodules or brain metastases, and then locally segment each small part independently. As a result, our method processes 3D medical images 50 times faster than DeepMedic and 3D U-Net with the same quality and requires 15 s or less on a CPU (see Figure 1). Our idea is simple and can be jointly applied with other acceleration techniques for additional acceleration and with other architecture improvements for quality increase. We have released (https://github.com/neuro-ml/low-resolution, accessed on 29 December 2020) our code for the proposed model and the experiments with the LUNA16 dataset to facilitate future research.

## 2. Related Work

Many different CNN architectures have been introduced in the past to solve the semantic segmentation of volumetric medical images. Popular models, such as 3D U-Net [11] or DeepMedic [10], provide convincing results on public medical datasets [7,18]. Here, we aim to validate the effective and well-known models without diving into architecture details. Following the suggestion of [19], we mainly focus on building a state-of-the-art deep learning pipeline arguing that the architecture tweaks will have a minor contribution. Moreover, we address that the majority of the architecture tweaks could be applied to benefit our method as well (see Section 7). However, all of these methods are based on 3D convolutional layers and have numerous parameters. Therefore, the inference time could be severely affected by the absence of GPU-accelerated workstations.

### 2.1. Medical Imaging

Several researchers have recently studied ways to accelerate CNNs for 3D medical imaging segmentation. The authors of [20] proposed a CPU-GPU data swapping approach that allows for training a neural network on the full-size images instead of the patches. Consequently, their approach reduces the number of iterations on training, hence it reduces the total training time. However, the design of their method can hardly be used to reduce the inference time. The design does not change the number of network’s parameters and moreover introduces additional time costs on the CPU-GPU data swapping process in the forward step alone. In [21], the authors developed the M-Net model for faster segmentation of brain extraction from MRI scans. The M-Net model aggregates volumetric information with a large initial 3D convolution and then processes data with a 2D CNN; hence, the resulting network has fewer parameters than the 3D analogies. However, the inference time on the CPU is 5 min for a 256×256×128 volume, which is comparable to that of DeepMedic and 3D U-Net. The authors of [22] proposed a TernaryNet with sparse and binary convolutions for medical image segmentation. It tremendously reduces the inference time on the CPU from 80 s for the original 2D U-Net [23] to 7 s. The additional use of proposed weight quantization can significantly reduce the segmentation quality. In general, 2D networks perform worse than their 3D analogous in segmentation tasks with volumetric images [11,24]. The simple and natural reason for it is the inability of 2D networks to capture the objects’ given volumetric information. Hence, in our work, we focus the attention on the 3D architectures.

Within our model, we use a natural two-stage method of regional localization and detailed segmentation (see Section 3). The similar approaches were proposed by [25,26]. In both papers, the first part of the method roughly localized the target anatomical region in the study. Then, the second part provided the detailed segmentation. However, the authors did not focus on the inference time and suggested using the methods to improve the segmentation quality. In addition, these architectures use independent networks, whereas we propose using weight-sharing between the first and second stages to achieve more effective inference. A similar idea of increasing the segmentation quality using multiple resolutions was studied by [27].

### 2.2. Nonmedical Imaging

The most common way to reduce the model size and thus increase inference speed is to use *mobile neural network architectures* [16], which are designed to run on low-power devices. For example, *E-Net* [28] replaces the standard convolutional blocks with asymmetric convolutions that could be especially effective in our 3D tasks. Additionally, *MobileNetV2* [29] uses inverted residual blocks with separable convolutions to reduce the computationally expensive convolutions. Unfortunately, most mobile architectures suffer from a loss in quality due to the achieved speed gain, which could be crucial for medical tasks.

## 3. Method

**Figure 2:**
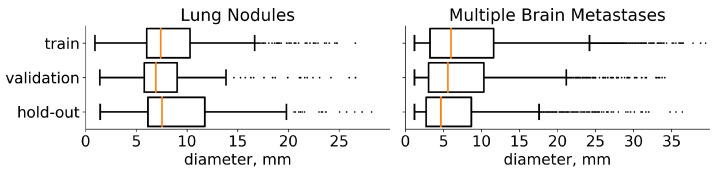
Diameter distribution of tumors in the chosen datasets. On both plots, the distribution is presented separately for each subset for which we split the data. The median value is highlighted with orange. In addition, medical studies [31,32] recommend choosing a 10 mm threshold for the data that contain lung nodules and 5 mm threshold for multiple brain metastases, when classifying the particular component of a target as small.

**Figure 3:**
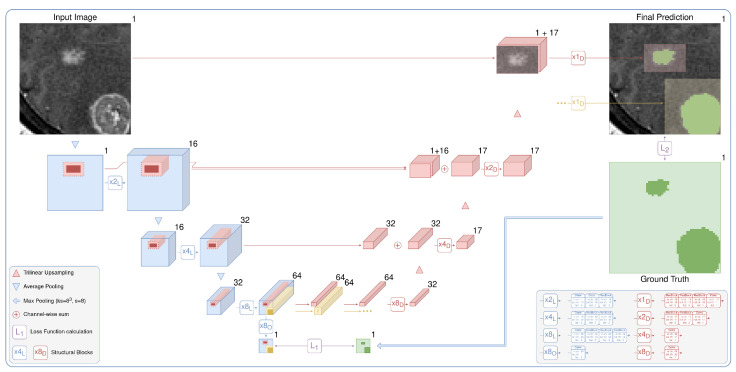
The proposed architecture is a two-stage fully convolutional neural network. It includes *low-resolution segmentation* (blue), which predicts the $8^{3}$ times downsampled mask, and *detailed segmentation* (red), which *iteratively* and *locally* aggregates features from the first stage and predicts the segmentation map in the original resolution. Speedup comes from two main factors: the lighter network with early downsampling in the first stage and the heavier second part that typically processes only 5% of the image.

The current state-of-the-art segmentation models spend equal computational resources on all parts of the input image. However, the fraction of the target on the images is often very low (e.g., $10^{-2}$ or even $10^{-3}$) for lung-nodule segmentation (see the distribution of the lesions diameters in the Figure 2). Moreover, these multiple small targets are distinct. With our architecture, *LowRes* (Figure 3), we aim to use the human-like approach to delineate the image. First, we solve the simpler task of *target localization*, modeling a human expert’s quick review of the whole image. Then, we apply *detailed segmentation* to the proposed regions, incorporating features from the first step. A similar idea was proposed in [30] where low resolution image was used to localize a large object prior to further segmentation to reduce GPU memory consumption.

We demonstrate the effectiveness of the method without delving into the architecture details. Our architecture is the de-facto 3D implementation of U-Net [23]. We use residual blocks (ResBlocks) [33], apply batch normalization, and ReLU activation [34] after every convolution except the output convolution. The number of input and output channels is shown for every convolution and ResBlock in the legend of Figure 3. We apply the sigmoid function to the one-channel output logits to obtain the probabilities of the foreground class. Note that our architecture could be trivially extended to solve the multiclass segmentation task by changing the number of output layers and replacing the sigmoid function with softmax.

### 3.1. Target Localization

The current approach to localizing targets is neural networks for object detection [35]. Nevertheless, classical object detection tasks often include multi-label classification and overlapping bounding box detection and are sensitive to a set of hyperparameters: intersection over union threshold, anchor sizes, and so on. We consider it to be overly complicated for our problem of semantic segmentation. Hence, we use the CNN segmentation model to predict the 8×8×8 times downsampled probability map (x8${}_{O}$ output in Figure 3). Processing an image in the initial resolution is the main bottleneck of the standard segmentation CNNs. We avoid this by downsampling an image before passing it to the model. For our tasks, we downsample an input image only twice in every spatial dimension. Applying downsampling with a factor of 4 gives us significantly worse results. We train the low-resolution part of our model via standard backpropagation, minimizing the loss function between the output probability map and the ground truth downsampled with 8×8×8 kernels of max pooling. It is detailed in Figure 3 in green.

Early downsampling and lighter modeling allow us to process a 3D image much faster. Moreover, the model can solve a “simpler” task of object localization with higher object-wise recall than the standard segmentation models. The U-Net-like architecture allows us to efficiently aggregate features and pass them to the next stage of detailed segmentation.

### 3.2. Detailed Segmentation

The pretrained first part of our model predicts the downsampled probability map, which we use to localize the regions of interest. We binarize the downsampled prediction using the standard probability threshold of 0.5. We do not use any fine-tuning of the threshold and also use this standard value to obtain a binary segmentation map for every model. After binarization, we divide the segmentation map into the connected components. Then, we create bounding boxes with a margin of 1 voxel (in low resolution) for every component. We apply the margin to correct the possible drawbacks of the previous rough segmentation step in detecting boundaries. The larger margins will sufficiently increase the inference time, hence we use the minimum possible margin.

The detailed part of our model predicts every bounding box in the original resolution. It processes, aggregates, and upsamples features from the first stage, similar to the original U-Net, but with two major differences: (i) the model uses features corresponding only to the selected bounding box and (ii) iteratively predicts every proposed component. The process is detailed in Figure 3 in red. The outputs are finally inserted into the prediction map with the original resolution, which is initially filled with zeros. Although the detailed part is heavier than the low-resolution path, it preserves the fast inference speed because it typically processes only 5% of the image.

We train the model through the standard backpropagation procedure, simply minimizing the loss function between the full prediction map and the corresponding ground truth. We use the pretrained first part of our model and freeze its weights while training the second part. The same training set is used for both stages to ensure no leak in validation or hold-out data, which could lead to overfitting.

## 4. Data

We report our results based on two large datasets: a private MRI dataset with multiple brain metastases and a publicly available CT chest scan from LUNA16 [17]. The dataset with multiple brain metastases consists of 1952 unique T1-weighted MRI of the head with a 0.94×0.94×1 mm image resolution and 211×198×164 typical shape. We do not perform any brain-specific preprocessing, such as template registration or skull stripping.

LUNA16 includes 888 3D chest scans from the LIDC/IDRI database [18] with the typical shape 432×306×214 and the largest at 512×512×652. To preprocess the image, we apply the provided lung masks (excluding the aorta component). In addition, we exclude all cases with nodules located outside of the lung mask (72 cases). Then, we clip the intensities to between −1000 and 300 Hounsfield units. We average four given annotations [18] to generate the mean mask for the subsequent training. Finally, we scale the images from both datasets to reach values between 0 and 1.

We use the *train-validation* setup to select hyperparameters and then merge these subsets to retrain the final model. The results are reported on a previously unseen *hold-out* set. Furthermore, LUNA16 is presented as 10 approximately equal subsets [17] so we use the first six for *training* (534 images), the next two for *validation* (178 images), and the last two as *hold-out* (174 images). Multiple metastases datasets are randomly divided into *training* (1250 images), *validation* (402 images), and *hold-out* (300 images). The diameters distribution of the tumors on these three sets for both datasets is given in Figure 2.

## 5. Experiments and Results

### 5.1. Training

We minimize Dice Loss [36] because it provides consistently better results on the validation data for all models. The only exception is the first stage of LowRes—we use weighted cross-entropy [23] to train it. We train all models for 100 epochs consisting of 100 iterations of stochastic gradient descent with Nesterov momentum (0.9). The training starts with a learning rate of $10^{-2}$, and it is reduced to $10^{-3}$ at epoch 80. During the preliminary experiments, we ensure that both training loss and validation score reach a plateau for all models. Therefore, we assume this training policy to be enough for the representative performance and the further fine-tuning will result only in the minor score changes.

We also use validation scores of the preliminary experiments to determine the best combination of patch size and batch size for every model. The latter represents a memory trade-off between a larger batch size for the better generalization and a sufficient patch size to capture enough contextual information. We set the patch size of 64×64×64 for all models except the DeepMedic. DeepMedic has a patch size of 39×39×39. The batch size is 12 for the 3D U-Net, 16 for DeepMedic, and 32 for the LowRes and mobile networks. Sampled patch contains a ground truth object with the probability of 0.5, otherwise it is sampled randomly. This sampling strategy is suggested to efficiently alleviate class-imbalance [10].

We use the DeepMedic and 3D U-Net architectures without any adjustments. Mobile network architectures [28,29] are designed for 2D image processing, hence we add an extra dimension. Apart from the 3D generalization, we use the vanilla E-Net architecture. However, MobileNetV2 is a feature extractor by the design, thus we complete it with a U-Net-like decoder preserving the speedup ideas.

### 5.2. Experimental Setup

We highlight two main characteristics of all methods: *inference time* and *segmentation quality*. We measure the average inference time for 10 randomly chosen images for each dataset. To ensure broad coverage of the possible hardware setups, we report time at 4, 8, and 16 threads on an Intel(R) Xeon(R) CPU E5-2690 v4 @ 2.60 GHz. Models running on a standard workstation have random-access memory (RAM) usage constraints. Hence, during the inference step, we divide an image into patches to iteratively predict them and combine them back into the full segmentation map. We operate under the upper boundary of 16 GB of RAM. We also noted that the inference time does not heavily depend on the partition sizes and strides, except the cases with a huge overlap of predicting patches.

The most common method to measure segmentation performance is the Dice Score [7]. However, averaging the Dice Score over images has a serious drawback in the case of multiple targets because large objects overshadow small ones. Hence, we report the average Dice Score per unique object. We use the Free-response Receiver Operating Characteristic (FROC) analysis [37] to assess the detection quality. Such a curve illustrates the trade-off between the model’s object-wise recall and average false positives (FPs) per image. The authors of [37] also extracted a single score from these curves—the *average recall* at seven predefined FP rates: 1/8, 1/4, 1/2, 1, 2, 4, and 8 FPs per scan. We report more robust values averaged over FP points from 0 to 5 with a step of 0.01. Hence, the detection quality is measured similarly to the LUNA16 challenge [17]. This is our main quality metric because the fraction of detected lesions per case is an important clinical characteristic, especially for lung-cancer screening [38].

### 5.3. Results

The final evaluation of the hold-out data of the inference time and segmentation quality is given in Table 1 for LUNA16 and in Table 2 for multiple metastase datasets. Moreover, LowRes achieves a comparable inference speed with the fastest 3D mobile network E-Net. The maximum inference time is 23 s with four CPU threads on LUNA16 data. Our model achieves the same detection quality using the state-of-the-art DeepMedic and 3D U-Net models, outperforming them in terms of speed by approximately 60 times. The visual representation of the time-performance trade-off is given in Figure 1.

## 6. Discussion

To our knowledge, the proposed method is the first two-stage network that utilizes local feature representation from the first network to accelerate a heavy second network. We carefully combined well studied DL techniques with the new final goal to speed up CPU processing time significantly without minor quality loss (see Section 1, the last paragraph). De-facto, our method can indeed be considered two separate networks as far as they are trained separately. However, we highlight that our work is one of the first accelerated DL methods for medical images, so it opens up a curious research direction of simultaneous learning of both CNN parts, among other ideas.

The “other ideas” mean the key concepts proposed to reduce the inference time or increase the segmentation and detection quality in many recent papers, which can be applied to our method. However, the research direction for medical image segmentation is naturally biased towards addressing the segmentation and detection quality, so these papers’ results are of particular interest. We can not address all of them here and provide comparison only to the well-known baseline methods like 3D U-Net and DeepMedic on the open-source LUNA16 dataset for the two main reasons. First, researchers can easily compare their great methods with ours and elaborate on this curious research direction. Second, most of the best practices from the recent methods can be adapted to ours without restriction. For example, one can modify skip connections by adding Bi-Directional ConvLSTM [39] or add a residual path with deconvolution and activation operations [40]. However, applying these improvements will likely preserve the time gap between the particular method and ours, modified correspondingly. Note that our pioneer work proposes the general idea of the speed up on CPU, so we treat such experiments as a possible way to work on in future, which is a common practice.

## 7. Conclusions

We proposed a two-stage CNN called LowRes (Figure 3) to solve 3D medical image segmentation tasks on a CPU workstation within 15 s for a single case. Our network uses a human-like approach of a quick review of the image to detect regions of interest and then processes these regions locally (see Section 3). The proposed model achieves an inference speed close to that of mobile networks and preserves or even increases the performance of the state-of-the-art segmentation networks (Table 1 and Table 2).

## Figures and Tables

**Table 1:** Comparative performance of segmentation models on LUNA16 data. Standard deviation for every measurement is given in brackets. The best values for each column are emphasized in bold. The quality metrics definitions are given in the last paragraph of Section 5.2.

	Inference Time * (CPU Threads)			Quality Metrics	
	4 Threads	8 Threads	16 Threads	Avg Recall	Obj DSC
3D U-Net	1293 (100)	880 (61)	828 (62)	**0.77** (0.02)	**0.82** (0.16)
DeepMedic	1139 (162)	872 (138)	840 (127)	**0.78** (0.02)	**0.78** (0.20)
3D MobileNetV2	108 (14)	89 (12)	74 (10)	0.68 (0.02)	0.75 (0.22)
3D E-Net	**11** (1.2)	**9.5** (1.2)	**9.2** (1.0)	0.62 (0.02)	0.70 (0.22)
LowRes	**23** (2.9)	**15** (1.9)	**13** (1.7)	**0.80** (0.02)	0.75 (.18)

* in seconds.

**Table 2:** Comparative performance of segmentation models on Multiple Metastases data. Standard deviation for every measurement is given in brackets. The best values for each column are emphasized in bold. The quality metrics definitions are given in the last paragraph of Section 5.2.

	Inference Time * (CPU Threads)			Quality Metrics	
	4 Threads	8 Threads	16 Threads	Avg Recall	Obj DSC
3D U-Net	342 (36)	225 (20)	202 (16)	**0.71** (0.01)	**0.72** (0.21)
DeepMedic	381 (74)	254 (50)	226 (46)	**0.70** (0.02)	**0.69** (0.23)
3D MobileNetV2	33 (4.6)	23 (3.0)	21 (2.4)	0.65 (0.01)	**0.69** (0.22)
3D E-Net	**3.2** (0.3)	**1.7** (0.2)	**1.9** (0.3)	0.47 (0.01)	0.59 (0.23)
LowRes	**6.1** (0.7)	**4.3** (0.6)	**3.7** (0.6)	**0.68** (0.01)	0.64 (0.22)

* in seconds.

## Data Availability

The CT chest scan dataset from LUNA16 competition presented in this study is openly available [17]. The MRI dataset with multiple brain metastasis presented in this study is not available publicly due to the privacy reasons.

## Abbreviations

The following abbreviations are used in this manuscript:

MDPI	Multidisciplinary Digital Publishing Institute
BraTS	Multimodal Brain Tumor Segmentation
CNN	Convolutional Neural Network
CPU	Central Processing Unit
CT	Computed Tomography
DSC	Dice Score
FP	False Positive
FROC	Free-response Receiver Operating Characteristic
GPU	Graphics Processing Unit
LUNA16	Lung Nodule Analysis 2016
MRI	Magnetic Resonance Imaging
RAM	Random Access Memory
ReLU	Rectified Linear Unit
